# Hypovolemic Phlebotomy in Hepatic Surgeries: Systematic Review and Updated Meta-Analysis of Blood Loss Reduction and Perioperative Outcomes

**DOI:** 10.7759/cureus.81879

**Published:** 2025-04-08

**Authors:** João G Oliva, Clara A Prado, Giovanna L de Assis, Guilherme A Figueiredo, Lucas V Ribeiro, Maria A Porto, Mariana F Duarte, Natália N Ribeiro, Victor F Silva, Elaine R Coelho

**Affiliations:** 1 Department of Life Sciences, State University of Bahia (UNEB), Salvador, BRA; 2 General Surgery, Escola Bahiana de Medicina e Saúde Pública (EBMSP), Salvador, BRA; 3 General Surgery, Salvador University (UNIFACS), Salvador, BRA; 4 General Surgery, UnidomPedro Afya, Salvador, BRA; 5 Digestive System Surgery, Roberto Santos General Hospital, Salvador, BRA

**Keywords:** blood loss, blood transfusion, hepatectomy, liver transplantation, phlebotomy, postoperative complications

## Abstract

Hepatic resection, especially in major procedures, is associated with high perioperative morbidity, primarily due to the significant risk of intraoperative hemorrhage and the consequent need for blood transfusions, factors that negatively impact clinical outcomes. Strategies to mitigate blood loss include hypovolemic phlebotomy (HP), a technique characterized by the controlled removal of a blood volume before liver transection, without immediate volume replacement, aiming to reduce central venous pressure (CVP) and consequently minimize intraoperative bleeding. Initial evidence suggests that HP may reduce the need for transfusions and improve the preservation of residual liver function, although its efficacy and safety still need to be validated in clinical studies with greater methodological robustness. This systematic review and meta-analysis aimed to assess the efficacy of hypovolemic phlebotomy in reducing blood loss during liver resections and improving perioperative outcomes.

This meta-analysis was conducted following the guidelines of the Preferred Reporting Items for Systematic Reviews and Meta-Analyses (PRISMA). To build the evidence base, a search was conducted in electronic databases, including PubMed, EMBASE, and the Cochrane Library, covering publications between 2010 and 2025. The risk of bias assessment was performed using RoB 2 for randomized clinical trials (RCTs) and ROBINS-I for observational studies. Statistical analysis was conducted using Review Manager 5.4. For binary variables, risk ratio (RR) was used, while for continuous variables, mean difference (MD) was applied in outcome analysis. Cochran’s Q test and Higgins’ I² were used to assess heterogeneity among studies.

Two RCTs and five observational studies were included, combining 3,369 patients undergoing liver surgery, with 1,759 (52.22%) in the HP group and 1,610 (47.78%) in the control group. HP was associated with a significant reduction in intraoperative blood loss (MD = -52.29; 95% CI -68.81 to -37.77; P < 0.00001). Additionally, there was a decrease in the need for intraoperative transfusion (RR = 0.27; 95% CI 0.19 - 0.38; P < 0.00001) and perioperative transfusion (RR = 0.43; 95% CI 0.28 - 0.66; P = 0.0001). Hospital length of stay showed no significant difference between groups (MD = -0.29; 95% CI -0.79 - 0.21; P = 0.26), nor did the analysis of major complications (Clavien-Dindo ≥ 3), which also did not demonstrate a statistically significant difference (RR = 0.94; 95% CI 0.72-1.24; P = 0.68).

In conclusion, HP demonstrated significant outcomes in liver surgeries, particularly in reducing intraoperative blood loss and the need for intraoperative and postoperative blood transfusions. Furthermore, the technique showed no significant difference in hospital length of stay, incidence of severe complications, or other clinical outcomes. Therefore, larger randomized studies are needed to determine the real impact of HP in different surgical settings.

## Introduction and background

Major hepatic resection is associated with significant perioperative morbidity, primarily due to the risk of excessive hemorrhage and consequent need for blood transfusions [1]. Despite advances in surgical, anesthetic, and intensive care techniques, these operations still present complications and continue to cause considerable intraoperative blood loss [2]. Studies indicate that blood loss during hepatectomy can vary significantly, ranging from 200 mL to 2000 mL, being an independent predictor of increased morbidity and responsible for up to 20% of deaths associated with hepatic resection [3,4].

Excessive blood loss and the consequent need for red blood cell (RBC) transfusions remain central concerns for surgeons, anesthesiologists, and patients [1]. In high-volume institutions, the RBC transfusion rate in patients undergoing hepatic resection ranges from 17% to 41% [5]. Although transfusions are potentially life-saving interventions, they are associated with risks such as transmission of infectious diseases, severe transfusion reactions, prolonged postoperative recovery, and worse long-term cancer-specific survival [4,5]. These factors reinforce the need for effective strategies to reduce blood loss and transfusion requirements in hepatic surgeries.

Hypovolemic phlebotomy (HP) emerges as a promising intervention in this context. This technique involves removing blood from the patient before hepatic transection begins, without immediate replacement by intravenous fluids, followed by retransfusion after resection [6]. The objective of this approach is to reduce circulating blood volume and portal flow, leading to a mild to moderate decrease in central venous pressure (CVP), which may result in less blood loss during surgery [4-6].

Additionally, HP has been associated with a significant reduction in hepatic venous pressure, which may decrease the risk of bleeding during hepatic parenchyma transection [4,5]. This technique may also contribute to preserving residual hepatic function, a critical factor for postoperative recovery, especially in patients with underlying liver disease or cirrhosis [6]. Despite these potential benefits, HP remains a technique requiring validation in robust clinical studies with larger samples and well-defined outcomes.

This study aims to systematically review the literature, including recent publications, updating the data on the effectiveness of hypovolemic phlebotomy in reducing blood loss and improving perioperative outcomes in hepatic resections. Additionally, it seeks to assess the impact of HP on reducing transfusion requirements, preserving liver function, and the incidence of postoperative complications, contributing to the optimization of clinical practices in this field.

## Review

Methodology

Search Strategy

The strategy used to build an evidence base for evaluating the outcomes of HP in liver surgeries was a systematic review with meta-analysis, guided by the latest Preferred Reporting Items for Systematic Reviews and Meta-Analyses (PRISMA) 2020 guidelines and registered in the International Prospective Register of Systematic Reviews (PROSPERO 2025) under the code CRD420250655359.

The PubMed, EMBASE, and Cochrane Library databases were used to identify studies covering the last 15 years of literature. Medical subject headings (MeSH/Emtree) were combined using Boolean operators (AND) and (OR): ("Hepatectomy" OR "Partial Hepatectomy" OR "Extended Hepatectomy" OR "Major Hepatectomy" OR "Minor Hepatectomy" OR "Liver surgery" OR "Liver resection" OR "Hepatic surgery" OR "Liver transplantation") AND ("Phlebotomy" OR "Bloodletting" OR "hypovolemic phlebotomy" OR "preoperative phlebotomy" OR "controlled hypovolemia" OR "hypovolaemic phlebotomy" OR "Intraoperative Phlebotomy").

Eligibility Criteria

Two reviewers independently assessed all retrieved studies according to the eligibility criteria, and any discrepancies between datasets were resolved by consensus or by a third reviewer. The included studies met the following criteria: randomized clinical trials, prospective or retrospective observational studies that directly compared the technique of hypovolemic phlebotomy with its omission in liver surgeries; original studies published in a peer-reviewed journal; studies involving adult patients (age >18 years); studies published between 2010 and 2025. The exclusion criteria focused on: insufficient data and/or the inability to calculate the outcomes; articles written in languages other than English; studies focused on pediatric patients; as well as narrative reviews, duplicate publications, and editorials.

Data Extraction

Two researchers performed the initial blinded duplicate screening by reading the following fields: title, keywords, and abstract. In this manner, the descriptors were required to appear in at least one of the three search fields (no limitation filters, such as article language, target population, or cutoff date, were applied), and subsequently, a full reading of the articles was carried out. The eligibility criteria for article inclusion and exclusion followed two stages: articles selected by both researchers were included; articles selected by only one researcher were analyzed by a third reviewer, who authorized inclusion for fitting. Data extraction was conducted independently and in duplicate by two reviewers using a standardized data collection form. Any discrepancies were resolved through discussion or adjudication by a third reviewer, thereby ensuring accuracy and consistency. The extracted variables comprised study title, first author, year of publication, study design, study period, total number of cases, postoperative complications, hospital length of stay, transfusion of red blood cell concentrates and other blood products, as well as intraoperative parameters.

Quality Assessment

The ROBINS-I tool was used to assess the risk of bias in non-randomized studies. This tool consists of seven domains that address biases related to confounding, participant selection, intervention classification, deviations from the intended intervention, missing data, outcome measurement, and selective reporting. Two reviewers independently assessed each domain, classifying the risk of bias as “low,” “moderate,” “serious,” or “critical” [7]. Disagreements were resolved by consensus, and the final classification followed the criteria established by the tool. For randomized clinical trials, the RoB 2 tool was applied. It consists of five domains that assess the risk of bias in the randomization process, deviations from the intended intervention, missing data, outcome measurement, and selective reporting. Two reviewers classified each domain as “low risk of bias,” “some concerns,” or “high risk of bias” [8]. Discrepancies were resolved by consensus, and the overall study assessment was determined according to the RoB 2 guidelines.

Statistical Analysis

The statistical treatment and graph production were performed with the aid of Review Manager 5.4 (RevMan) software, starting with the organization of data extracted into tables in Microsoft Excel (Redmond, WA, USA). The overall results were represented by weighted mean, accompanied by the ± weighted standard deviation (SD). The statistical analysis of the data obtained from the studies focused on relative risk/risk ratio (RR) for dichotomous variables and mean difference (MD) for continuous variables, considering a confidence interval of 95% (p < 0.05) as statistically significant. Cochran’s Q test and the I² statistic were executed to evaluate the heterogeneity among the studies included in the review. An I² value < 25% indicated low heterogeneity among the studies; 25% < I² < 50% indicated moderate heterogeneity among the studies; and I² ≥ 50% indicated a high degree of heterogeneity among the studies [9]. To account for unexplained heterogeneity and adjust for variability, the random-effects model was selected using the Mantel-Haenszel method. A subgroup analysis was performed for outcomes with sufficient data to constitute subgroups. Outcomes with incomplete data were not included in the graphical synthesis. Articles in which continuous variables were represented by the median, with their measure of dispersion being range or interquartile range, had their results estimated for mean and standard deviation [10,11].

Results

Study Selection

**Figure 1:**
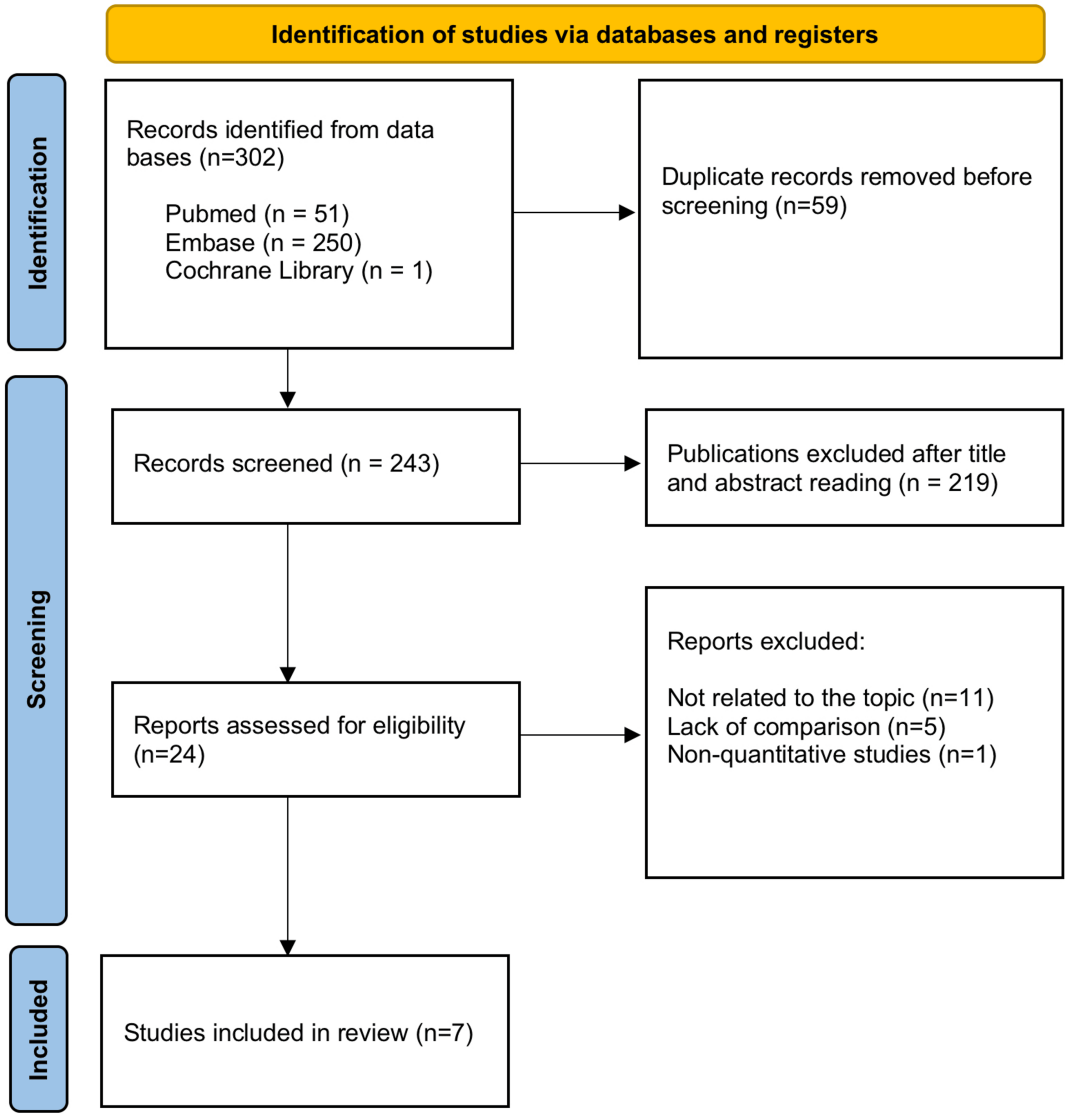
Flowchart of article selection according to Preferred Reporting Items for Systematic Reviews and Meta-Analyses (PRISMA)

A total of 302 studies were found in the initial search in the indexing databases: 51 in PubMed, 250 in Embase, and one in the Cochrane Library. Fifty-nine duplicate studies were excluded before screening, leaving 243 for evaluation. Two hundred nineteen publications were excluded based on the title and abstract review. Twenty-four publications were examined through full-text reading, and seven references were classified for inclusion in the study. The search process is summarized in the flowchart in Figure 1.

The combined analysis involved 3369 patients who underwent liver surgery, with 1759 (52.22%) forming the intervention group, in which hypovolemic phlebotomy was performed, and 1610 (47.78%) forming the control group. The total sample used in the statistical synthesis consisted of 2101 (62.36%) male individuals and 1268 (37.64%) female individuals. The average age of patients in the hypovolemic phlebotomy group was 51.92 (±11.52) years, and in the control group, it was 57.03 (±12.10) years. The average volume of blood removed during the procedure was 410.41 (±121.46) mL. The characteristics of the included studies are presented in Table 1.

**Table 1:** Studies included in the systematic review

Author name	Year of publication	Study model	Study period	Hypovolemic phlebotomy	Control
						Rekman et al. [12]	2017	Prospective observational	2013 to 2016	37	101
Baker et al. [13]	2019	Retrospective observational	2010 to 2016	45	316
Martel et al. [14]	2020	Randomized controlled trial	2016 to 2018	31	31
Khaldi et al. [15]	2021	Retrospective observational	2011 to 2017	522	161
Carrier et al. [16]	2022	Retrospective observational	2008 to 2021	365	314
Massicotte et al. [17]	2022	Retrospective observational	2002 to 2019	536	464
Martel et al. [18]	2025	Randomized controlled trial	2018 to 2023	223	223
			Total	1759	1610

Risk of Bias Assessment

**Figure 2:**
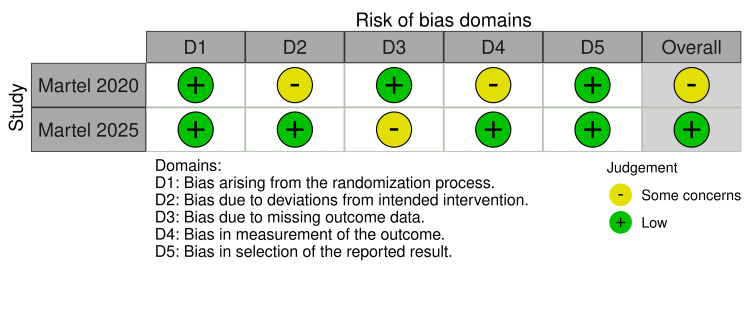
Risk of Bias Assessment using Risk of Bias 2 (RoB 2) Martel 2020 [14]; Martel 2025 [18].

The assessment of the methodological quality of the studies included in this review was performed using the RoB 2 tool for randomized clinical trials and the ROBINS-I tool for cohort-type observational studies. Two clinical trials were evaluated using RoB 2 (Figure 2), and the results indicated a difference in the risk of bias between the studies. One of the clinical trials showed a low risk of bias in all domains assessed, including the randomization process, deviations from the intervention, missing outcome data, outcome measurement, and selection of reported outcomes. This study was therefore classified as having a low overall risk of bias. On the other hand, the second clinical trial showed some concerns in the domains related to bias due to deviations from the intended intervention and bias in outcome measurement. Although it presented a low risk of bias in the other domains, the presence of these limitations resulted in an overall classification of some concerns regarding the risk of bias.

**Figure 3:**
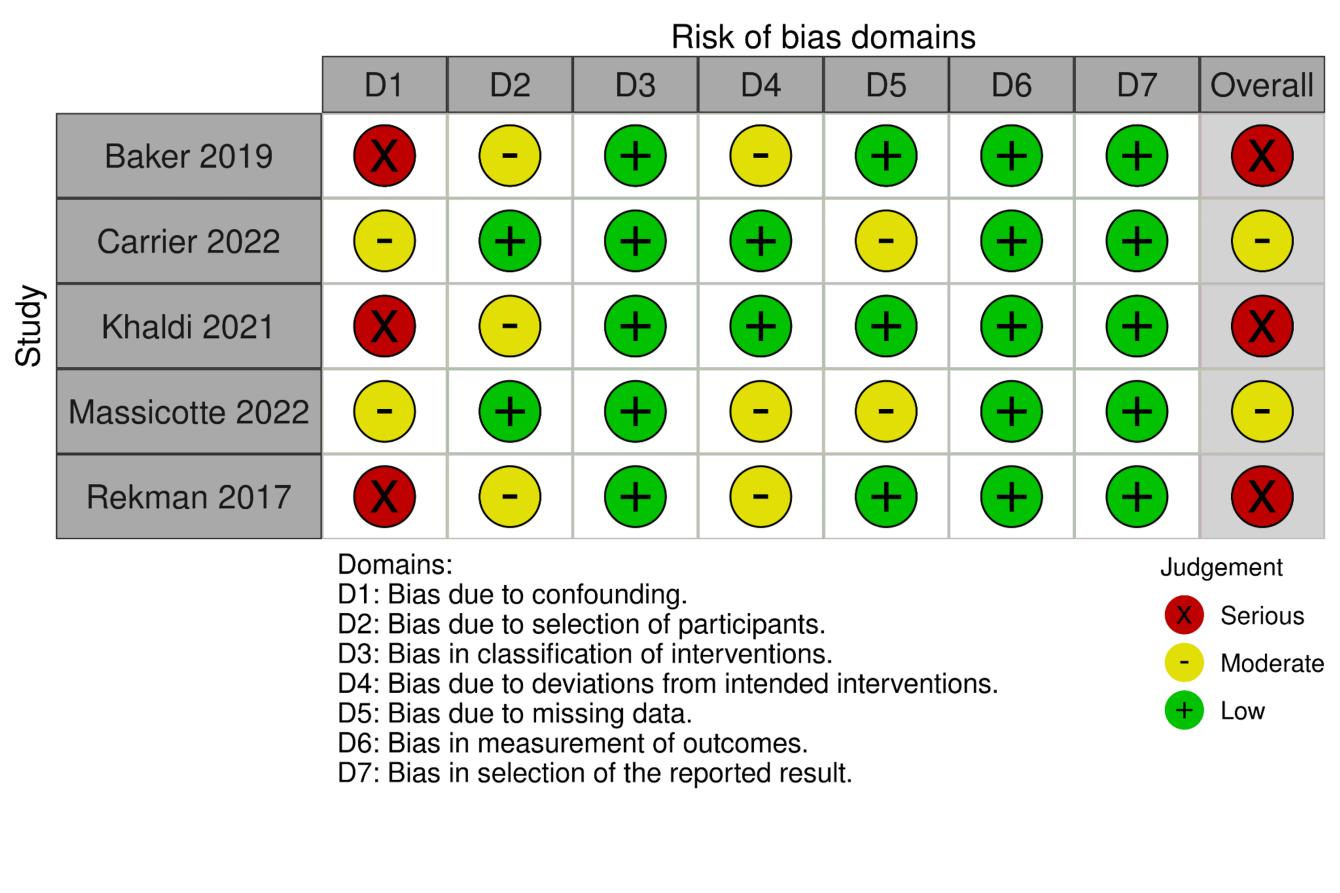
Risk of Bias Assessment using Risk of Bias in Non-Randomized Studies of Interventions (ROBINS-I) Baker 2019 [13]; Carrier 2022 [16]; Khaldi 2021 [15]; Massicotte 2022 [17]; Rekman 2017 [12].

The analysis of the five observational studies was conducted using the ROBINS-I tool (Figure 3), which revealed significant variations in the risk of bias among the studies. Three studies were classified as having a serious risk of bias, primarily due to issues in the domains of confounding bias and participant selection bias. The remaining two studies showed a moderate risk of bias, with concerns focused on participant selection bias and outcome measurement bias.

Primary Outcomes

**Figure 4:**
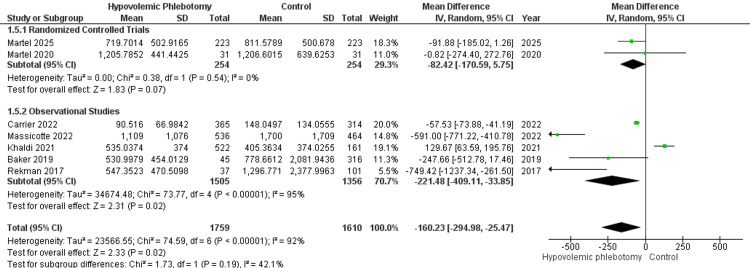
Forest plot of blood loss in the intraoperative period between hypovolemic phlebotomy (intervention) and control Martel 2025 [18]; Martel 2020 [14]; Carrier 2022 [16]; Massicotte 2022 [17]; Khaldi 2021 [15]; Baker 2019 [13]; Rekman 2017 [12].

The volume of blood lost during the intraoperative period was assessed in seven studies, two randomized clinical trials (RCT; 508 participants) and five observational studies (OS; 2861 participants), combining 3369 patients in the statistical synthesis. A subgroup analysis was performed based on the types of studies: RCT (MD = -82.42; 95% CI -170.59 - 5.75; P = 0.07) and OS (MD = -52.36; 95% CI -68.12 - -36.59; P < 0.00001). The overall analysis demonstrated a statistically significant reduction in blood loss with the hypovolemic phlebotomy technique compared to the control group (MD = -52.29; 95% CI -68.81 - -37.77; I² = 92%; P < 0.00001; Figure 4) with high heterogeneity among the studies.

**Figure 5:**
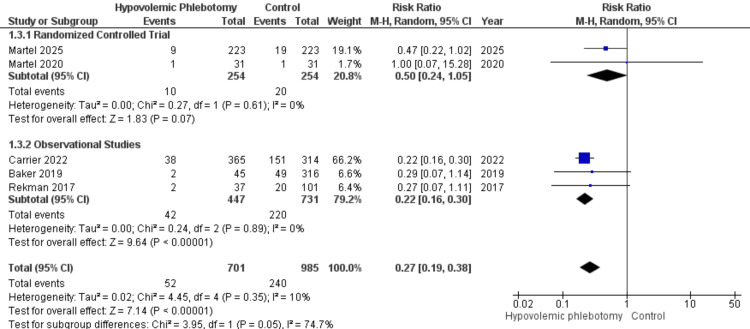
Forest plot of the number of intraoperative transfusions between hypovolemic phlebotomy (intervention) and control Martel 2025 [18]; Martel 2020 [14]; Carrier 2022 [16]; Baker 2019 [13]; Rekman 2017 [12].

The number of red blood cell transfusions during the intraoperative period was examined in five studies, assessing the outcome in 1686 participants, of whom 701 underwent HP and 985 did not. Subgroup analysis was performed based on study design: RCT (RR = 0.50; 95% CI 0.24 - 1.05; P = 0.07) and OS (RR = 0.22; 95% CI 0.16 - 0.30; P < 0.00001). The combined statistic demonstrated a significantly lower association of HP with the number of intraoperative transfusions compared to its non-performance (RR = 0.27; 95% CI 0.19 - 0.38; P < 0.00001; I² = 10%; Figure 5) with low heterogeneity among the studies.

**Figure 6:**
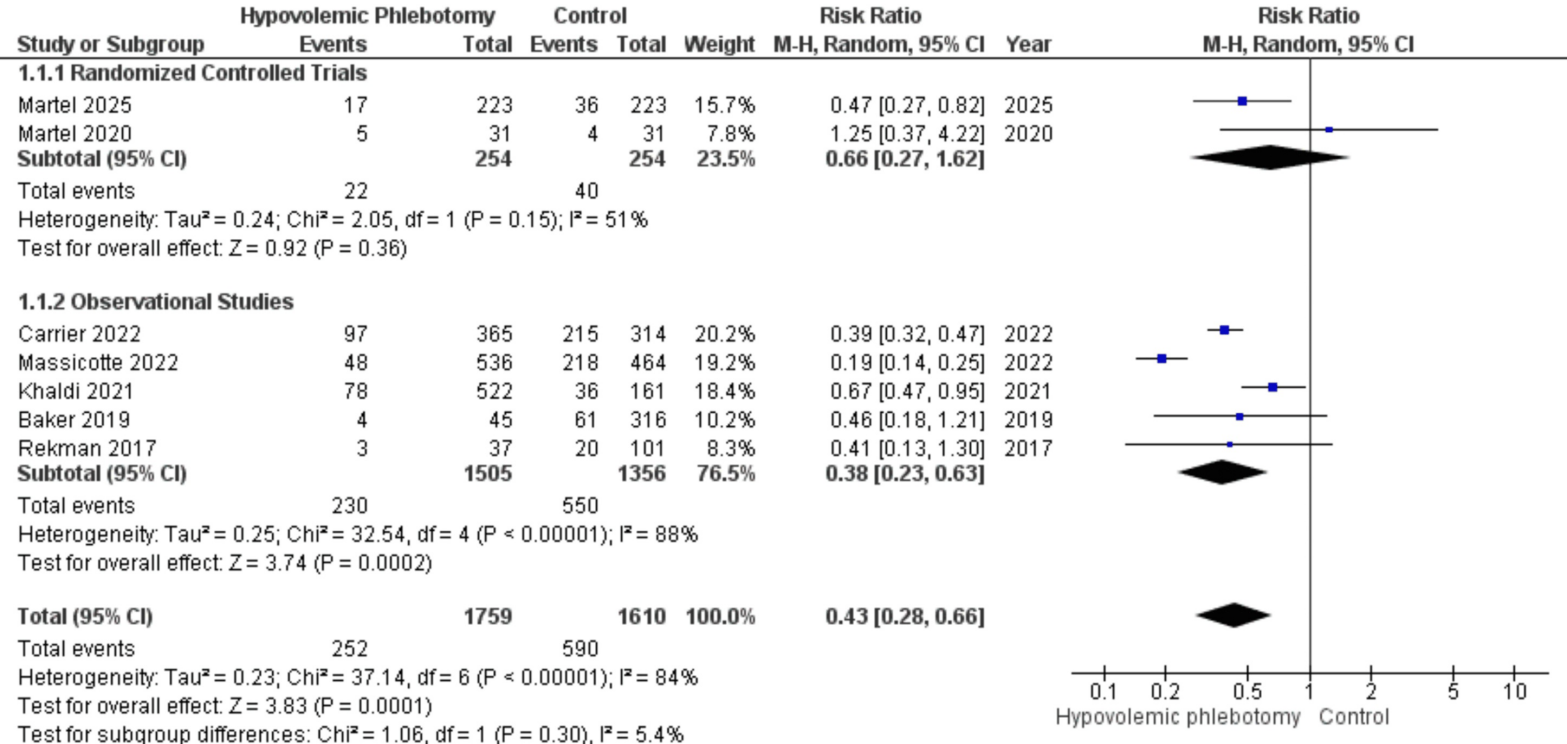
Forest plot of the number of red blood cell transfusions in the perioperative period between hypovolemic phlebotomy (intervention) and control Martel 2025 [18]; Martel 2020 [14]; Carrier 2022 [16]; Massicotte 2022 [17]; Khaldi 2021 [15]; Baker 2019 [13]; Rekman 2017 [12].

Seven studies assessed the occurrence of transfusions during the perioperative period of liver surgeries, involving 1610 patients who underwent HP and 1759 in the control group. In the subgroup analysis, the subtotal of randomized clinical trials did not show a statistically significant reduction in the outcome due to the intervention (RR = 0.66; 95% CI 0.27 - 1.62; P = 0.36). However, the observational studies (RR = 0.38; 95% CI 0.23 - 0.63; P = 0.0002; I² = 88%), as well as the overall result (RR = 0.43; 95% CI 0.28 - 0.66; P = 0.0001; I² = 84%), revealed a statistically significant difference. Despite this, the high heterogeneity observed limits the reliability of the findings, as shown in Figure 6.

Secondary Outcomes

**Figure 7:**
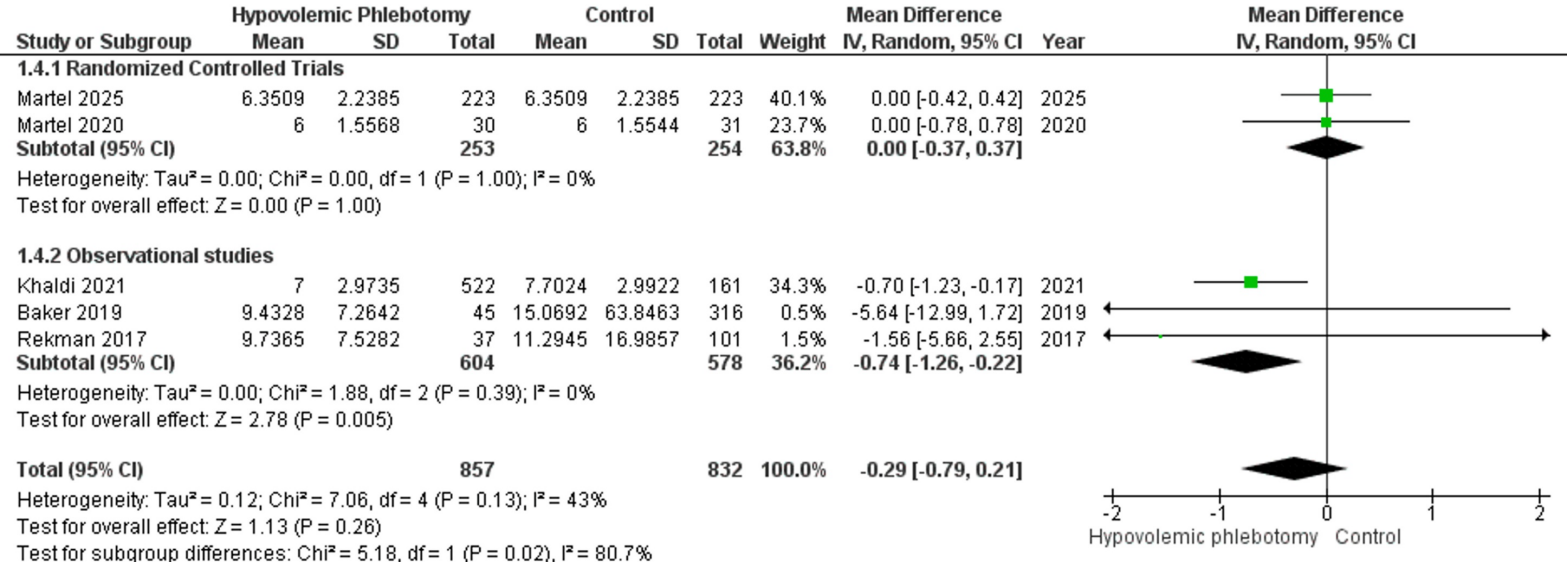
Forest plot of the hospitalization time analysis between patients undergoing hypovolemic phlebotomy (intervention) and control Martel 2025 [18]; Martel 2020 [14]; Khaldi 2021 [15]; Baker 2019 [13]; Rekman 2017 [12].

Hospitalization time was studied by five references, grouping 1689 individuals for statistical synthesis (Figure 7). Among the randomized clinical trials, no statistically significant difference was found between performing the procedure or not (MD = 0.00; 95% CI -0.37 - 0.37; P = 1.00; I² = 0%). Observational studies demonstrated a reduction in the number of days of hospitalization (MD = -0.74; 95% CI -1.26 - -0.22; P = 0.05; I² = 0%), in contrast to the overall analysis, where no significant association was found for the outcome in patients undergoing hypovolemic phlebotomy (MD = -0.29; 95% CI -0.79 - 0.21; P = 0.26; I² = 43%).

**Figure 8:**
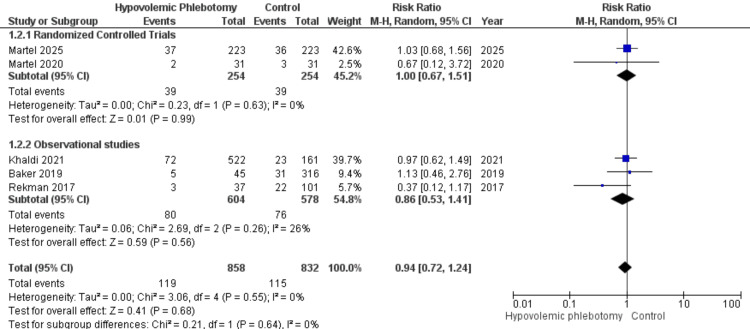
Forest plot of the analysis of the number of major postoperative complications between patients undergoing hypovolemic phlebotomy (intervention) and control Martel 2025 [18]; Martel 2020 [14]; Khaldi 2021 [15]; Baker 2019 [13]; Rekman 2017 [12].

Five references studied the occurrence of major complications (Clavien-Dindo ≥ 3), two randomized clinical trials (RR = 1.00; 95% CI 0.67 - 1.51; P = 0.60; I² = 0%) and three observational studies (RR = 0.86; 95% CI 0.53 - 1.41; P = 0.26; I² = 26%), totaling 1690 patients in the combined statistical synthesis. The overall analysis showed an RR of 0.94 (95% CI 0.72-1.24; P = 0.68; I² = 0.68). Therefore, there is no statistical evidence that hypovolemic phlebotomy significantly modifies the outcome analyzed (Figure 8).

Discussion

This study systematically reviewed the existing literature on HP, a technique used during liver resection that involves the controlled removal of blood before liver transection, without immediate volume replacement. This approach aims to reduce CVP, minimizing intraoperative blood loss and the need for red blood cell transfusions. According to the European Association for the Study of the Liver (EASL) guidelines [19], intraoperative strategies for managing blood loss, including HP, are recommended to improve surgical outcomes in patients undergoing liver resection. In line with this, it is known that major liver surgeries are associated with high perioperative morbidity, primarily due to the risk of significant hemorrhage and the consequent need for blood transfusions [20].

This meta-analysis demonstrated the impact of HP on intraoperative blood loss and perioperative outcomes in patients undergoing liver resection. A total of 3369 patients undergoing liver surgery were analyzed, with 52.22% in the intervention group, which received HP, and 47.78% in the control group. The sample included 2101 men (62.36%) and 1268 women (37.64%). The findings suggest that the technique is effective in reducing the volume of blood lost during the procedure (MD = -52.29; 95% CI -68.81 - -37.77; I² = 92%; P < 0.00001), as well as significantly reducing the need for red blood cell transfusions. These results are consistent with previous studies highlighting the relationship between reduced CVP and improved hemostatic control [13].

HP has proven to be an effective strategy in reducing intraoperative blood loss in high-risk liver resections. In the PRICE-2 RCT, the median blood loss was significantly lower in the intervention group (679 mL, IQR 400-1074 mL) compared to the control group (800 mL, IQR 481-1152 mL), demonstrating a substantial difference in intraoperative hemorrhage [18]. This technique, which involves the removal of 7-10 mL/kg of total blood before liver transection without volume replacement, resulted in a reduction in the need for red blood cell transfusions, reinforcing its clinical applicability in minimizing complications associated with hemorrhage and allogeneic transfusions. These findings support the hypothesis that HP improves surgical conditions and contributes to the optimization of perioperative safety, making it a viable approach for high-complexity hepatic procedures [18].

In contrast, intraoperative hemorrhage and the need for red blood cell transfusions remain significant challenges in liver surgery, despite contemporary advancements in surgical and anesthetic techniques. Current guidelines for the intraoperative management of patients undergoing liver resection present various strategies aimed at reducing blood loss and transfusion requirements. However, most of these recommendations are based on limited evidence, such as inferior vena cava clamping, terlipressin infusion, and volume replacement guided by stroke volume variation, which have been evaluated in small-scale RCTs. These studies demonstrated an impact on the estimation of intraoperative blood loss, but without robust evidence of clinical benefits [20].

The reduction in the perioperative RBC transfusion requirement is particularly relevant, as transfusions are associated with worse prognoses in liver surgeries, including a higher risk of infectious complications, immunosuppression, and a negative impact on long-term survival in oncological patients. The analysis of postoperative blood transfusion in the PRICE-2 study showed that HP significantly reduced the need for red blood cell transfusions after surgery. Therefore, the meta-analysis demonstrates that HP significantly reduces the transfusion rate of blood components and was well accepted by surgeons, who reported better visibility and control of the surgical field [16,17]. Nevertheless, the high heterogeneity of the studies supports the idea that more RCTs are needed to elucidate the safety and effectiveness of this technique.

The analysis of hospitalization time in the present study revealed that HP was not associated with a significant reduction in hospital stay duration when considering RCTs (MD = 0.00; 95% CI -0.37 - 0.37; P = 1.00). However, observational studies showed a tendency for a decrease in this scenario (MD = -0.74; 95% CI -1.26 - -0.22; P = 0.05). These findings align with the study by Carrier et al. (2022), where the substantial difference in hospital stay between patients who underwent HP and those treated conventionally was null [16]. Nonetheless, the PRICE-2 study (Martel et al., 2025) suggested that the reduction in transfusion requirements may be related to a more favorable postoperative recovery, indirectly impacting the length of stay [18].

Regarding major complications, it was analyzed that there is no statistical evidence that HP significantly alters the outcome. These findings are consistent with the studies analyzed. The analysis of major complications (Clavien-Dindo ≥ 3) showed that HP did not significantly increase the risk of severe adverse events [15]. This result reinforces the safety of the technique, suggesting that controlled hypovolemia induction does not compromise the hemodynamic stability of patients, as long as it is applied in a judicious manner.

The results of the PRICE-2 RCT demonstrated that the incidence of severe complications (≥ grade 3a on the Clavien-Dindo classification) was similar between the intervention group (17%) and the control group (16%), with no statistically significant difference (RR 1.06; 95% CI 0.70-1.61), indicating that the technique does not increase the risk of severe adverse events. However, an increase in the incidence of grade A biliary fistula was observed in the group undergoing HP (16% vs. 9% in the control group; adjusted RR 1.71; 95% CI 1.02-2.85), although this complication is generally self-limiting and of lower clinical impact [18]. These findings suggest that HP should be applied with caution, considering the patient’s risk profile and the potential incidence of biliary complications.

Limitations of the study

Despite the promising results, several limitations must be considered. First, the high heterogeneity observed in the outcomes of blood loss (I² = 92%) and perioperative red blood cell transfusion (I² = 84%) suggests that factors such as variations in surgical techniques, anesthetic protocols, blood volume removed, and transfusion criteria may influence the results. Furthermore, the predominance of retrospective observational studies raised serious concerns regarding the presence of confounding bias and moderate concerns about selection bias, which may have affected the measurement of outcomes. Additionally, there was inconsistency in the classification of Clavien-Dindo ≥ 3 complications, with some studies not distinguishing between grade IIIa and IIIb, and others starting the categorization at grade IV. Further RCTs with larger sample sizes are needed to confirm these findings and establish more robust guidelines for the use of hypovolemic phlebotomy in liver surgeries.

## Conclusions

In conclusion, hypovolemic phlebotomy demonstrated significant outcomes in terms of reducing intraoperative blood loss, as well as the need for intraoperative and postoperative blood transfusions in liver surgeries. As a result, it is evident that this technique can help minimize exposure to blood transfusions, thereby reducing associated potential risks. Additionally, no statistically significant difference was observed regarding the impact of HP on hospitalization time and the incidence of severe complications. Thus, while HP has shown promise in reducing blood loss and the need for blood transfusions during the intra- and postoperative periods, its influence on other clinical outcomes remains uncertain. Therefore, further randomized studies with larger sample sizes and assessment of outcomes in various scenarios are needed to demonstrate its true impact in surgical practice.
